# A Reverse Modification Method for Binary Code and Data

**DOI:** 10.3390/s22207714

**Published:** 2022-10-11

**Authors:** Lei Yu, Yucong Duan

**Affiliations:** 1 Department of Computer Science, Inner Mongolia University, Hohhot 010021, China; 2 Department of Computer Science, Hainan University, Haikou 570228, China

**Keywords:** reverse engineering, 3D graphics, virtual reality, assembly code

## Abstract

This paper reveals the hidden dangers of reverse data modifications on distributed software with network synchronization, during the era of 5G, which may occur in more important domains, such as telemedicine and automatic driving. We used pseudo-codes to formally elaborate the distributed software architectures and design patterns. It is necessary to deal with three challenges for the modification of binary code and data in the distributed software architectures: (1) the base virtual addresses of software objects are changed frequently for safety; (2) prior knowledge of the reverse is not considered; (3) system memory values of some target objects are changed with extreme speed. For this purpose, a novel reverse modification method for binary code and data is proposed. According to the knowledge-based rules, our method can manipulate physical data, sight data, animation data, etc., while the game synchronization mechanism cannot detect the changes. The implementation details of our method are presented using high-level programming languages (C++) and low-level programming languages (assembly), based on multiple snippets, so that readers can understand both the overall distributed software developments and the corresponding reverse processes. In particular, two network games are used for the demonstrations in this paper. The demonstration results show that our proposed methodology is efficient (as proved by formulas and practices) to manipulate the codes and data of distributed software using a synchronization mechanism.

## 1. Introduction

Human knowledge is constantly updating and affects any human-designed system. Although deep learning techniques have simulated human brains to automatically extract features, this does not mean that a deep learning system can fulfill the tasks of reverse engineering without human knowledge [1]. On the contrary, if human knowledge can be fused with reverse engineering, it will accelerate the speed of reverse engineering. There is plenty of necessary knowledge [2] by which to decode the machine codes successfully. From this perspective, software reverse engineering represents knowledge engineering about disassembly or decompilation. To deal with the problems related to this paper, human knowledge provides help for reverse engineering in a forward-thinking way. 

Two typical games are selected for the experiments: a real-time strategy game (RTSG) and a first-person shooting game (FPS). The two games are also used for experiments and validations because simulated environments have no correlation with real game environments in terms of huge virtual addresses, real stack traces, function import tables, etc. This paper solves one major problem: what the essential knowledge is in software engineering for accelerating reverse engineering, and how we can use it.

There are three challenges in the problem:

1. The base virtual addresses of software objects can be changed frequently for safety, leading to invalid access to old addresses found by reverse methods. The same reverse process is repeated once the base virtual addresses of software objects change. For example, nearly every month, software providers can patch local files and change the base addresses through the internet.
2. Prior knowledge of reverse is not considered; thus, a more efficient reverse process cannot be invented to reduce the processing time. However, it is a challenge to fuse prior knowledge to reverse engineering, to accelerate the processing speed.
3. Huge objects are generated in the software, and the values of some target objects are changed with extreme speed; thus, it is hard to completely obtain whole values from outside processes. In addition, the player list in an FPS is consecutive in the system memory. Unlike an FPS, the game objects list in an RTSG is not consecutive in the system memory.

To tackle the above challenges, a novel reverse method is proposed for manipulating class members based on knowledge, called MK (manipulation based on knowledge), which is presented in Section 3, Section 4 and Section 5. The main novelty and contributions of this paper are identified as follows:

1. A new methodology (MK) is proposed to reverse-manipulate class members, based on knowledge in three-dimensional (3D) video games, and its reverse procedure is automatic and intelligent. MK is effective when dealing with challenge 1 and challenge 2 in this paper. Assembly codes are injected into the target software to obtain the filtered values from outside processes, which is effective for dealing with challenge 3 in this paper.
2. The general architectures of multi-player online 3D video games are presented via high-level program codes (the forward perspective) and disassembly codes (the reverse perspective). The program slicing of game logic, the rendering pipeline, and data synchronization are presented.

The motivation and purpose of the work are to draw the researchers’ attention to the fact that this domain should be studied further because reverse data manipulation for distributed software will shortly become important in the 5G era. Specifically, the novelties of our work include: (1) explaining the meaning of “seeing is believing” for distributed software with network synchronization, (2) how to achieve “seeing is not believing”, and (3) where and when to reverse-manipulate the data. For demonstrating these new hidden dangers, the detailed reverse process is elaborated in real scenarios.

This paper is organized as follows: Section 2 discusses software analysis and knowledge-related methods. Section 3 presents general architectures and design patterns of 3D network games from a developer’s point of view. Section 4 constructs a knowledge base for a reverse engineer or an attacker to manipulate the data in general software domains. Most importantly, according to computer graphics theory, Section 5 introduces how to cheat the network synchronization mechanisms for displaying the additional information that is usually hidden by game producers. Section 6 presents a demonstration to prove that “seeing is not believing” in network games if an attacker holds the knowledge that we present in Section 3, Section 4 and Section 5.

## 2. Related Works

As Table 1 shows, a literature review was conducted to present the state-of-the-art and knowledge gaps of previous research with relevance to the topic of this paper.

Whatever high-level program source codes or disassembly codes are obtained, the dependency relations of target variables can usually be discovered [3]. The dependency relations of target variables include control flow and data flow. The dependency relations of a target variable can be obtained by static and dynamic program slicing [4]. To reduce the number of dependency relations, dynamic program slicing uses specific inputs while static program slicing does not. The disadvantage of program slicing is that program codes must have previously been obtained. 

Symbolic executions [5,6] are technologies that analyze control flow or extract path constraints, which are used to find all feasible program paths and detect information leakage [7]. Jeong et al. used fast and flexible deep learning via the symbolic graph execution of imperative programs [8]; program paths can be predicted with progressive symbolic execution [9]. However, symbolic execution technologies are not scalable for large programs with unbounded loop iterations [10,11]. 

Taint analysis [12] are technologies to analyze program information flow to detect vulnerabilities or malware. Dynamic taint analysis has been applied to android systems [13,14]. For boosting taint analysis, Chen et al. [15] used dynamic taint analysis with neural networks. Nevertheless, the problem of hidden taint propagation restricts the development of taint analysis, to a certain extent [16].

Because deep learning programs can possess knowledge [17] and basic inference ability [18,19], the following research about inference has been studied. Glatt [20] proposed the deep case-based policy inference algorithm to accelerate learning by building a library of cases and reusing them if they are similar to a new task when training a new policy. The algorithm guided the training by dynamically selecting and blending policies according to their usefulness for the current target task, reusing previously learned policies for a more effective exploration but still enabling adaptation to the particularities of the new task. Kumar [21] observed that caching intermediate layer outputs can help avoid running all the layers of a DNN for a sizeable fraction of inference requests [22]. They proposed approximate caching (in different domains [23]) at each intermediate layer.

Wu [24] introduced a human-in-the-loop “rubric sampling” approach to tackle the “zero-shot” feedback challenge. Deep learning inference enables the further improvement of rubric sampling as more specific data are acquired; Kang [25,26] considered that deep learning inference often needs to be performed locally in distributed environments. Predicting the remaining useful life of machinery, infrastructure, or other equipment can facilitate preemptive maintenance decisions, whereby a failure can be prevented through timely repair or replacement. Kraus [27] proposed a neural network for predicting a system’s remaining useful life, which combines the favorable properties of these approaches: its key innovation is that it offers both high accountability and the flexibility of deep learning [28]. The parameters are estimated via variational Bayesian inferences. 

The previously mentioned research intends to use the interior inferences of deep neural networks, which are slow in situations where we have explicit knowledge or rules. Therefore, the inference mechanisms of explicit knowledge or rules in deep neural networks were studied. Formal methods promise to expand the use of state-of-the-art learning approaches in the direction of certification and sample efficiency. Hasanbeig [29] proposed a deep reinforcement learning (RL) method for policy synthesis in continuous-state/action in unknown environments, under the requirements expressed in linear temporal logic (LTL). A modular deep deterministic policy gradient (DDPG) architecture is proposed to generate a low-level control policy that maximizes the probability of the given LTL formula. Leon [30] presented a neuro-symbolic agent that combines deep reinforcement learning with temporal logic and achieves systematic out-of-distribution generalization in tasks that involve following a formally specified instruction. Specifically, the agent learns the general notions of negation and disjunction and successfully applies them to previously unseen objects without further training. 

Gauthier [31] proposed a deep reinforcement learning framework based on self-supervised learning within the proof assistant, HOL4 (high-order logic). A close interaction between the machine learning modules and the HOL4 library is achieved by the choice of tree neural networks (TNNs) as machine learning models and the internal use of HOL4 terms to represent the tree structures of TNNs. A Monte Carlo tree search (MCTS) algorithm, guided by a TNN, can be used to explore the search space and produce better examples for training the next TNN. Hosny [32] proposed a reinforcement learning-based methodology that navigates the optimization space without human intervention. The training of an advantage actor-critic (A2C) agent seeks to minimize the area subject to a timing constraint. Wang [33] proposed to integrate logical knowledge in the form of first-order logic into a deep learning system, which can be trained jointly in an end-to-end manner. The integrated framework is able to enhance neural outputs with knowledge regularization via logic rules, and at the same time update the weights of logic rules to comply with the characteristics of the training data. 

Deep Logic Models [34] created an end-to-end differentiable architecture, where deep learners are embedded into a network implementing the continuous relaxation of the logic knowledge. The learning process allows jointly learning the weights of the deep learners and the meta-parameters controlling high-level reasoning. The predicates and functions of first-order logic knowledge can be bound to any TensorFlow computational graph [35], then the formulas can be converted into a set of real-valued constraints, which participate in the overall optimization problem. This allows learning the weights of the learners, under the constraints imposed by prior knowledge. By combining first-order logic or high-order logic, recursive neural networks have used special activation functions, but it still needs a great deal of time to compute the gradients and propagate backward. 

## 3. Dissecting Code Blocks and Data Structures

The essential knowledge in MK is described as “rules” and is translated to program codes from this section. If the software requirements are determined, the common features of the corresponding implementations for requirements can be guessed. Thereafter, the guess regarding the features can be confirmed by an analysis of the disassembly codes. Based on C++ programming on the Windows platform, we constructed the following simplified codes (blend C#, C++, and Python style) to illustrate several parts of the architecture in RTS and FPS games. Notice that: (1) an indention style is used so that all identifiers {} are removed, to be more concise; (2) all accesses of class members are public. 

### 3.1. Game Architecture and Main Loop

There are basically three models for game logic: a one-thread loop, a multi-thread loop, and a jobs loop. Fast computers and slow computers have different numbers of floating-point operations during the same elapsed time, which leads to different round-off errors and causes a world state of non-synchronization among the various computers. Therefore, network games update their components in fixed time and render them in variable time. The following game architecture (Algorithm 1) uses one thread to update a physics component, an animation component, an AI component, etc., in 16 ms, and renders an image in variable time, which depends on the rendering workload. In addition, it uses a non-blocking socket to send and receive synchronization data.
**Algorithm 1:** Main loop in a one-thread scenario.lag = 0 Ioctlsocket(socket) // non-blocking while (! quitGame) InputHandle()  currentTime = GetCurrentTime() elapsed = currentTime–lastTime lastTime = currentTime  lag += elapsed while Lag >= 16 ms   UpdateGameObjects()   UpdateAnimations()   CollisionDetect()   Physics()   Ragdoll()   GenerateFinalPoses()   AI()   EventSystem.Dispatch()   lag− = 16 ms Render (lag/16)

**Figure 1:**
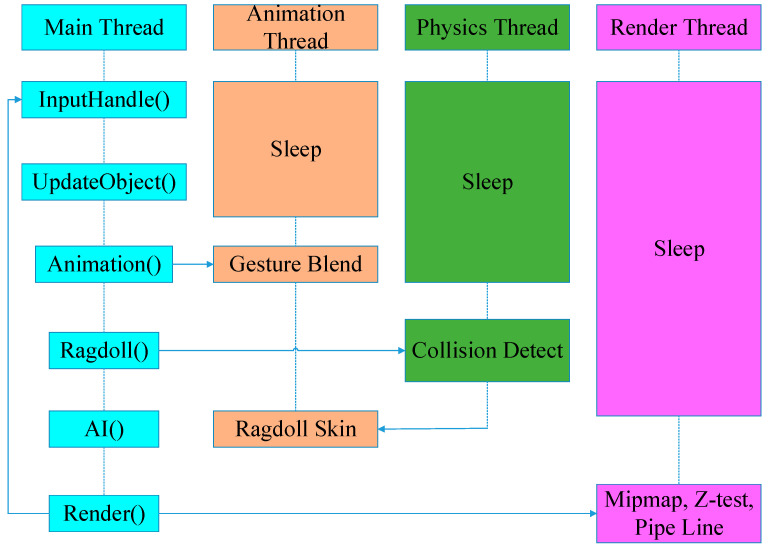
The architecture for multiple threads.

Large 3D video games use multiple threads to interact with the main thread (Figure 1). Each thread implements several special functions that communicate with other threads. The thread rendering pipeline is used to draw game objects to the local screen. Moreover, an independent thread for network data synchronization can be used to verify whether the game data is modified in the multiplayer online environment. 

When the CPU cores increase, the job model is more scalable because the scheduler in the job model eliminates sleep time, as shown in the multi-thread model above. 

### 3.2. Decrease the Probability of Guessing Wrongly by Decompiling

How do we know what architecture is used in software? Decompiling software is one way to establish this, but this is an NP (Non-deterministic Polynomial time) problem. Decompiling technology can produce readable (although sometimes not fully understandable) and high-level languages, which help us decrease the probability of guessing wrongly what software architectures and design patterns are used by the developers, even though few tools can restore high-level program codes perfectly. The following codes (Algorithm 2) are partially decompiled codes.
**Algorithm 2:** Decompiled codes.int __thiscall a3_fun(unsigned __int8 *this, int a2, _DWORD *a3) {  _DWORD *v3; // edi  unsigned __int8 *v4; // esi  bool i; // cf  unsigned __int8 v6; // al  int v7; // ecx  char v8; // bl  int result; // eax  int v10; // ecx  int v11; // [esp+Ch] [ebp-78h]  __int64 v12; // [esp+18h] [ebp-6Ch]  int v13; // [esp+20h] [ebp-64h]  int v14; // [esp+80h] [ebp-4h]  v3 = a3;  v4 = this;  for ( i = a3[2] < (unsigned int)(*a3−a3[1]); i; i = v3[2] < (unsigned int)(*v3−v3[1]))  {    if ( *(_DWORD *)v4 <= 1u )    {      if ( !*(_DWORD *)v4 )        break;      v6 = v4[4];      if ( !v6 )        break;      *(_DWORD *)v4 = 0;      v4[4] = v6 - 1;    }    sub_143FB80(&a3);  } …  return a2_ fun (a2 + 16, v3); } 

### 3.3. Game Object

Decompiling is not ideal for compiling languages such as C++, as shown above. Therefore, we used our skills as game developers to write the following snippet codes in the paper. The snippet codes are accurate explanations that will work even better than natural language descriptions because codes contain accurate syntax and semantic information, which is important for accelerating reverse engineering. For example, to efficiently update the game components and avoid missing CPU caches, the following architecture (Algorithm 3) and design patterns are those that are most commonly used in games.
**Algorithm 3:** GameObject with components.Class GameObject   Vector3 position, translate, rotate, scale   Mesh mesh   Material * materials   Texture * textures   int num_materials   InputComponent * input   PhysicsComponent *physics   GraphicsComponent *graphics   void Update()      input -> Update (this)      physics -> Update (this)      graphics -> Update (this) Class InputComponent   virtual void Update (GameObject & go)      if IsPressed(LEFT) go.position.x++ Class GraphicsComponent   // Mesh, Textures, Shaders   virtual void Update (GameObject & go)      if go. position.x < 50 Draw (IMAGE_TRAP) Class PhysicsComponent   // rigidbody, mass   virtual void Update (GameObject & go)      ResolveCollisions(go. position.x) Class AIInputComponent: InputComponent   //use AI to replace human players   virtual void Update (GameObject & go)      ObserveWorldState()      TakeActions() Class Player   List<CombatUnit> BuiltCUs   CombatUnit GetCombatUnit(TYPE type, ID id) Partial Class CombatUnit: GameObject   int sight, rank   float health   List<CombatUnit> ObjectDetect() {//return detected objects}   virtual int ExpandSight()      if m_Health > 0          sight = sight * rank       else         sight = 0      return sight Class T70: CombatUnit   virtual int ExpandSight()       if health > 0          sight = sight * 2       else         sight = 0      return sight int Game_Logic(Player player)      CombatUnit * pCombatUnit = player.GetCombatUnit(T70, 1)      pCombatUnit –>ExpandSight()       List<CombatUnit> detectedObjects = pCombatUnit –>ObjectDetect()

At the bottom of the snippet, the video game RTSG is used as a scenario. Here, a player has a list of combat units that are built during the game. T70 is a Soviet tank that is derived from the class CombatUnit. ExpandSight() is a virtual function that is implemented separately by the class CombatUnit and its subclass T70. Because of run-time polymorphism, function T70::ExpandSight() will override the function CombatUnit::ExpandSight(). The disassembly would be “call eax”, not a specific address. Polymorphism is one of the most powerful mechanisms that object-oriented programs bring to code reuse. The base classes can call upon the methods of derived class instances without recompiling, while maintaining the interfaces unchanged. These invocations will demand more time to analyze the program flow because we need to find the virtual function table first.

An annotation tree or an abstract syntax tree can be automatically generated from snippet codes of high-level programming language by the existing tools. The annotation tree or the abstract syntax tree contains enough semantic information to be used to build a series of rules. These rules can adapt to software changes, such as DLL files update, interface deletion, logic revision, and variable name revision.

## 4. Disassemble Online Video Games from Machine Codes in Reverse Perspective

Most of the time, disassemblers produce accurate disassembly codes from the machine codes, and this is where most reverse data modifications happen. The essential knowledge used by MK to accelerate reverse engineering is constructed in this section and can be extracted as rules. 

### 4.1. Mapping the Virtual Address to the File Offset Address

When the target data are found in the memory, its virtual address (VA) is needed to map to the file offset address (FOA) to patch the file stored in the hard disk (Algorithm 4). Every executable file (.exe, .dll, .sys, and .com, etc.) in Windows systems uses a PE (portable executable) structure. The PE structure has an “Image_Optional_Header”, which includes “SectionAlignment” and “FileAlignment”, indicating data alignment in the memory and hard disk.
**Algorithm 4:** Manipulating data in the memory and files.// Relative Virtual Address (RVA) = VA – ImageBase if sectionAlignment == fileAlignment  dataFOA = dataRVA else  dataFOA = sectionFOA + (dataRVA – sectionRVA )

### 4.2. Searching Based on Code Features

We simulated the Microsoft Visual C++ Compiler (MSVC 14.0) x86 to write the following assembly codes. MSVC increasingly prefers SSE (streaming SIMD (single instruction, multiple data) extension) instructions instead of FPU (float point unit) instructions to operate floating-point data. Therefore, health information is obtained by the instruction “movss” (move scalar single-precision floating-point) and is then stored in the 128-bit register, xmm0, in SSE. The following snippet (Algorithm 5) shows the high-level codes and the possible disassembly codes.
**Algorithm 5:** Reverse ExpandSight().Class T70: CombatUnit virtual int ExpandSight()     if health > 0       sight = sight * 2     else      sight = 0    return sight Corresponding disassembly codes:  push ebp   mov ebp, esp  push esi    mov esi, ecx  mov eax, [esi+4]  movss xmm0, DWORD PTR [esi+C]  comiss xmm0, 0  jna @loc1  imul eax, 2   jmp @loc2  @loc1:   mov eax, 0   @loc2:     mov [esi+4], eax   pop esi    pop ebp  ret          

Prior knowledge about game logic is important, especially when a game updates and patches its codes frequently. If attackers know that the game developer often uses Sight * 2 (C++ programming) to expand the sight of a unique combat unit, the attackers may find out the address of the sight by searching the byte array of imul eax, 2 (the corresponding assembly codes) in several seconds. However, the same game logic can be implemented by equivalent assembly codes if compiler optimization is used. These equivalent assembly codes perform the same task, but they are executed faster from a CPU perspective. For example, the assembly code imul eax, 2 can be replaced by the assembly code shl eax, 1. The assembly code mov eax, 0 can be replaced by the assembly code xor eax, eax. Moreover, attackers also need to know the byte order of the system so that byte-array searching can return the right results. In this case, number 2 is stored in the memory as 0x00000002 in the “big-endian” system, while 0x02000000 in the “little-endian” system. If they consider this factor, attackers are also able to find the right answer at an acceptable speed. 

### 4.3. Calling Conventions

Other information should be constructed as knowledge before and after a function is invoked. Generally, compilers push function parameters into the system memory stack in a right-to-left order before a function is invoked (push 1 then push address @T70). There are some differences after a function is invoked, whether the caller function or callee function will clean the stack and pop arguments of the invoked function: in other words, calling conventions. CDECL, STDCALL (WINAPI), and FASTCALL are standard calling conventions that are related to both reverse analysis and code patching. The object of the class is often stored in ecx, while the return value of a function is stored in eax or xmm0. In our examples (Algorithm 6), STDCALL is used (add esp or retn).
**Algorithm 6:** Reverse GameLogic().int Game_Logic(Player player)  CombatUnit *pCombatUnit = player.GetCombatUnit(T70, 1)  pCombatUnit –>ExpandSight()   List<CombatUnit> detectedObjects = pCombatUnit –>ObjectDetect() Corresponding disassembly codes:  push ebp  mov ebp, esp  sub esp, 8  push 1  push @T70   lea ecx, DWORD PTR [ebp+8]  call @GetCombatUnit   mov [ebp-4], eax  mov eax, DWORD PTR [ebp-4]  mov edx, DWORD PTR [eax]  lea ecx, DWORD PTR [ebp-4]  call edx   call @ObjectDetect  mov DWORD PTR [ebp-8], eax   add esp, 8  pop ebp  retn 4

Polymorphism mechanisms increase the difficulties of reverse engineering because reverse engineers need to identify whether the object of the base class or the object of the derived class is using the function when they step into a virtual function in debug mode. Compilers set up a virtual function table for each related class, to resolve function calls in a dynamic binding manner. Therefore, reverse engineers may look up virtual function tables; they need to know whether the virtual table pointer might be stored in eax, while the first address of ExpandSight() might be stored in edx. 

### 4.4. Disabling Target Function

A target function may appear in multiple places in the whole program. If the target function needs to be disabled, nop can be patched on it in each relevant place. However, the arguments and the stack also need to be cleaned, making the process complicated. Alternatively, when bypassing the target function, the calling convention of the target function should be discovered first. Then, the first opcode in the target function can be replaced by retn x, where x/4 indicates the number of arguments. If STDCALL is used, we replace the first opcode with retn x (0xC2); otherwise, if CDECL is used, we replace the first opcode with retn (0xC3).

### 4.5. Filtering out Unrelated Addresses in Identified Memory Areas

Address space layout randomization (ASLR) is a security technique that is primarily used in operating systems to protect against buffer overflow attacks. ASLR can put game program addresses (image base, initial stack, heap, PEB, etc.) in unpredictable locations. However, the target data addresses must be in specific memory areas: data, stack, or heap. Target code addresses must be in a specific memory area: .text. Neither of them must be in the kernel space. 

### 4.6. Searching Based on Initial Settings

The game’s initial settings are important factors by which to accelerate the search speed. If attackers already know that one anti-tank gun causes 160 points of damage to a tank from the front armor, attackers will allow the tank to get the shot and then directly search the value (current_health_tank – 160), thereby identifying the address of health_tank. Similarly, positive directions of screen coordinates can be used as prior knowledge to accelerate coordinate searching. OpenGL uses right-handed Cartesian coordinates, while Direct3D uses left-handed Cartesian coordinates.

### 4.7. Resources Allocation

The arrangement of resources in the memory is the primitive information leakage source. When “malloc” and “new” are used, the heap allocator will automatically change from user mode to kernel mode and produces a memory buffer, which is slow. Hence, some games use their memory allocators, as follows (Algorithm 7).
**Algorithm 7:** Active array.Class ActiveObjects   GameObject objects[n] // keep data together for cache line, avoid pointer chasing   int firstInactive // no need to load the GameObject in the right of firstInactive void ActiveAnObject(int i)    Swap(objects[firstInactive], objects[i])    firstInactive ++ void DeactiveAnObject(int i)    firstInactive --    Swap(objects[firstInactive], objects[i])

Similar to AddRef() in the Windows COM, when resources (e.g., textures, vertex buffers) are allocated in the memory, their reference counters are begun to avoid frequent loading and unloading from the memory. Hence, attackers cannot manipulate those resources where the reference number is zero. The pool allocator can divide the memory into sequential and small chunks of the same volume to speed up the loading and unloading process and avoid memory fragments (Algorithm 8).
**Algorithm 8:** Free list.Class Soldier: GameObject  Union      Struct { float x, y, z }     Soldier * next Class Pool Soldier soldiers[n] // sequential and small chunks, avoid fragment Soldier * firstAvailable  // increase flexibility at the same time void ActiveAnObject()    Soldier * newsoldier = firstAvailable   firstAvailable = newsoldier ->getNext()   newsoldier ->initial(x,y,z) void DeactiveAnObject(int i)    soldiers [i].setNext(firstAvailable)   firstAvailable = & soldiers [i]

## 5. Manipulating “Seeing” by MK

Based on enough knowledge, as mentioned above for MK, attackers can manipulate data (including vision data, animation data, physics data, event data, etc.) to change the software’s intrinsic behaviors (including how to present game objects, simulate a physical world, etc.).

### 5.1. Seeing Is Not Believing 

“Seeing is not believing” means that software users can see objects that the software does not intend to display; meanwhile, the software believes that the objects are not displayed. Section 4 presents the knowledge used in general software, while subsequent sections present the knowledge used in multiplayer online games.

### 5.2. Manipulating Vision Data in the Local Computer

“Seeing” can be manipulated by changing the data in the rendering process. The rendering process includes matrix transform, back-face culling, occlusion culling, etc. 

#### 5.2.1. Where Is a Bad Place for Injection?

There are improper places in the rendering process to inject codes to change the data. D3D 11 supports multi-threads to parallel-create resource objects (e.g., textures) by generating a command list. In addition, “compute shader” can be used for computing tasks by the GPU, instead of the CPU. Now, programmers can develop shader programs run in GPU by high-level shader language, which is an “Effect” file (.fx). GPU obtains the vertex data in the system memory from the vertex buffer filled by the CPU, while the GPU uses functions such as SetMatrix to transmit variables in the system memory to the graphic memory. Therefore, codes and data in the GPU should be untouched to avoid “hardcore mode”, while there are proper places to inject codes, to change the data with minimum effort, as the following methods show.

#### 5.2.2. A Fast but Inaccurate and Unreliable Method

There are over 1000 game objects (soldiers, tanks, bunkers, etc.) in RTSG that need to be monitored in real time. The following injection codes are used to obtain the visual range (rectangle: lx = 28h, lz = 20h) of the camera in a fast but inaccurate way. The rationale is that an axis-aligned bounding box (AABB) is used to represent the visual range where any game object is inside, as Algorithm A1 in Appendix A shows.

The space transforms are in this order: model space, world space, camera space, projection space (also known as homogeneous clip space), and screen space. Because the coordinate data can be obtained by reverse engineering, the transforms are constructed by attackers in this order: world space, camera space, projection space, and screen space. 

The original point of the camera in world coordinates is o. Here, *x*, *y*, and *z* are axes, while *u*, *v*, and *w* are the right vector, up vector, and forward vector, respectively, in the world coordinates, then the transform matrix from camera coordinates to world coordinates is:

(1) $$T_{camera2world}=\left[\begin{array}{cc} \begin{array}{cc} u_{x} & u_{y}\\ v_{x} & v_{y} \end{array} & \begin{array}{cc} u_{z} & 0\\ v_{z} & 0 \end{array}\\ \begin{array}{cc} w_{x} & w_{y}\\ o_{x} & o_{y} \end{array} & \begin{array}{cc} w_{z} & 0\\ o_{z} & 1 \end{array} \end{array}\right]$$

Thus:

(2) $$T_{world2camera\;}\overset{@1}{↔}{\;T}_{camera2world}^{-1}\;\overset{@2}{↔}\;{\left(\mathrm{RT}\right)}^{-1}\;=T^{-1}R^{-1\;}\overset{@3}{↔}{\;T}^{-1}R^{T}=\left[\begin{array}{cc} \begin{array}{cc} 1\; & 0\\ 0\; & 1 \end{array} & \begin{array}{cc} 0 & \;0\\ 0 & \;0 \end{array}\\ \begin{array}{cc} 0 & 0\\ -o_{x} & -o_{y} \end{array} & \begin{array}{cc} 1\; & 0\\ -o_{z} & 1 \end{array} \end{array}\right]\;\left[\begin{array}{cc} \begin{array}{cc} u_{x} & v_{x}\\ u_{y} & v_{y} \end{array} & \begin{array}{cc} w_{x} & 0\\ w_{y} & 0 \end{array}\\ \begin{array}{cc} u_{z} & v_{z}\\ 0 & 0 \end{array} & \begin{array}{cc} w_{z} & 0\\ 0 & 1 \end{array} \end{array}\right]$$

Annotation @1: The transform matrix from camera coordinates to world coordinates is an inverse transform matrix from world coordinates to camera coordinates. 

Annotation @2: The transform matrix from camera coordinates to world coordinates can be obtained by a translation matrix T and a rotation matrix R.

Annotation @3: The rotation matrix is an orthogonal matrix.

Range of x’, y’ and z’ in projection space is [−1.0, 1.0], [−1.0, 1.0], and [0.0, 1.0] respectively. Assuming that a coordinate (x’, y’) in the projection plane is projected by a point (x, y, and z) in camera space, then (x’, y’) can be obtained by:

y’ = d y/z = y cotangent (fov/2)/z (3)

The distance between the projection plane and the original point is d, and the field of vision is “fov”. Likewise, x’ = x cot(fov/2)/z. We change the range of x’ to [−1.0, 1.0], x’ = x cot(fov/2) / z r, where aspect ratio is r. We change the range of z’ from the near plane and far plane [n, f] to [−1.0, 1.0], and we assume that z’ = A z + B. Thus, a projection matrix can be obtained by a scale matrix and a translation matrix:

(4) $$T_{camera2projection}=\left[\begin{array}{cc} \begin{array}{cc} \cot\left(\mathrm{fov}/2\right)/r & 0\\ 0 & \cot\left(\mathrm{fov}/2\right) \end{array} & \begin{array}{cc} 0 & 0\\ 0 & 0 \end{array}\\ \begin{array}{cc} 0\; & 0\\ 0\; & 0 \end{array} & \begin{array}{cc} A & 1\\ B & 0 \end{array} \end{array}\right]$$

(5) $$\left[x,y,z,1\right]\left[\begin{array}{cc} \begin{array}{cc} \frac{\cot\left(\frac{\mathrm{fov}}{2}\right)}{r} & 0\\ 0 & \cot\left(\frac{\mathrm{fov}}{2}\right) \end{array} & \begin{array}{cc} 0 & 0\\ 0 & 0 \end{array}\\ \begin{array}{cc} 0\; & 0\\ 0\; & 0 \end{array} & \begin{array}{cc} A & 1\\ B & 0 \end{array} \end{array}\right]=\left[\frac{\cot\left(\frac{\mathrm{fov}}{2}\right)x}{r},\frac{\cot\left(\frac{\mathrm{fov}}{2}\right)y}{r},\mathrm{Az}+B,z\right]$$

Divide z:

(6) $$\left[\frac{\cot\left(\frac{\mathrm{fov}}{2}\right)x}{\mathrm{zr}},\frac{\cot\left(\frac{\mathrm{fov}}{2}\right)y}{\mathrm{zr}},A+B/z,1\right]$$

Map z so that n-> 0 and f-> 1:

(7) $$\left\{\begin{array}{c} A+\frac{B}{n}=0\\ A+\frac{B}{f}=1 \end{array}\right.\;\overset{}{\Rightarrow }\left\{\begin{array}{c} A=\frac{f}{f-n}\\ B=\frac{-nf}{f-n} \end{array}\right.$$

(8) $$T_{projection2viewport\;}=\left[\begin{array}{cc} \begin{array}{cc} w/2 & 0\\ 0 & -h/2 \end{array} & \begin{array}{cc} 0 & 0\\ 0 & 0 \end{array}\\ \begin{array}{cc} 0\; & 0\\ w/2\; & h/2 \end{array} & \begin{array}{cc} 1 & 0\\ 0 & 1 \end{array} \end{array}\right]$$

Finally transforms $T_{world2camera\;},{\;T}_{camera2projection}\;,{\;T}_{projection2viewport\;}$ are multiplied by attackers to display game objects on their screens.

#### 5.2.3. A Fast and Accurate but Unreliable Method

**Figure 2:**
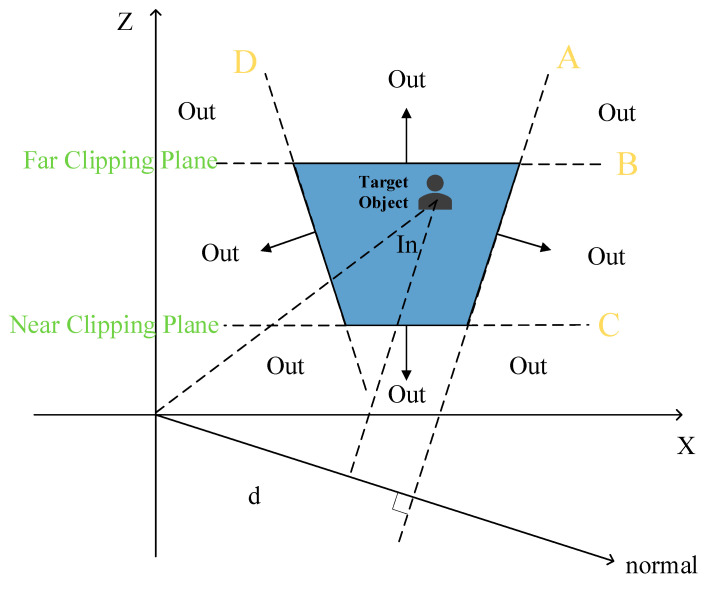
The range of camera sight.

Different from the above method, the actual shape of the visual range in RTSG is a trapezoid rather than a rectangle (shown in Figure 2) because the “look vector” of the camera is diagonally downward at the height of the *y*-axis. The local player cannot see any object outside of the trapezoidal area that is surrounded by lines A, B, C, and D, which separate the space into pieces (depending on the player’s camera). 

**Figure 3:**
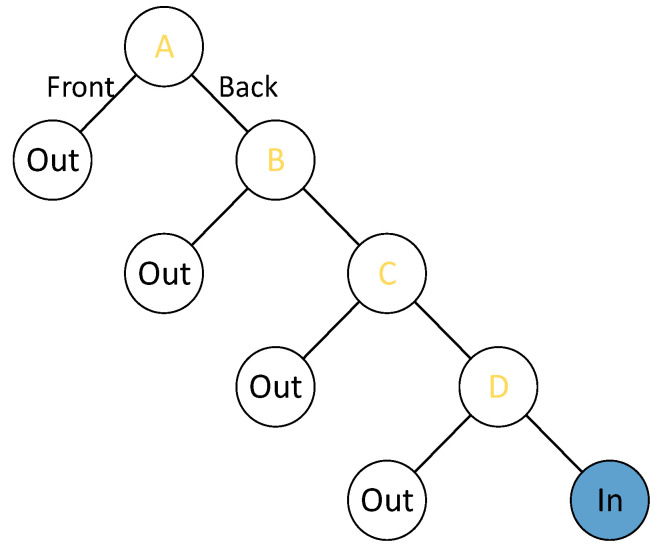
BSP tree.

In the trapezoidal scenario (Figure 2), BSP (binary space partition) can be used to rapidly retrieve target objects, while most of the objects are outside the visual range. Both Figure 3 and Algorithm A2 in Appendix A describe the retrieval process. The objects outside the trapezoid will be displayed in the mini-map; meanwhile, the objects inside the trapezoid will be displayed in the main window of the local player.

There are three kinds of rotation that can be considered when camera data are manipulated. The matrix rotation uses 16 memory units, while the Euler rotation uses only three. However, the Euler rotation is an unreliable method. If the rotation axis is the world axis, the order of rotation determines the final rotation result; that is, different rotation orders will ultimately lead to different rotation results. The Euler rotation may cause an object to lose a certain direction of rotation (the degree of freedom), which is a gimbal lock, meaning that no matter how we rotate in this state, it is impossible to achieve the desired rotation effect unless we break the original rotation order or rotate three axes at the same time. The reason is that matrix multiplication does not satisfy the commutative law; thus, a quaternion rotation is better for interpolation. 

#### 5.2.4. A Fast, Accurate and Reliable Method

Related matrices can be obtained directly from the system memory, but certain tricks are needed. These tricks are not revealed in this paper, to protect game development. 

#### 5.2.5. Extended Methods

Other techniques can be used to manipulate seeing. For example, smoke simulation in games can be implemented by the CPU or geometry shader in the GPU, then a z-buffer and stencil buffer are used to change the frame buffer. In this case, the billboard and particle system are weak points; moreover, oriented bounding boxes in the collision detection module can be exploited to achieve invincibility.

### 5.3. Manipulating Vision Data for Cooperative Seeing

As we described above, it is feasible to enable extrasensory data for a single client. Moreover, it is effective to use a composition system, to enable more extrasensory data on multiple clients in a cooperative way.

The real application of this process in multiple clients is in the area of cooperative cheating. For example, m_iDormant is a boolean in FPS that determines whether the player information is updated or not. If a network player is close enough to a local player, even when behind walls, the FPS server will set a false value for the m_iDormant of the network player and send the local player information about the network player. If the network player is far away from the local player, the server will not send updated information about that player (m_iDormant is true). 

If a teammate_C of the local player_A kills enemy_B, who is dormant for local player_A, and the local client tries to read the health of the killed enemy_B, the game will show that enemy_B is still alive, even though teammate_C has just killed enemy_B. However, once the local player_A gets closer to the dead enemy_B, the correct information will be updated at that time.

Therefore, a player only sees “nearby” related data from the game servers. The solution of cooperative seeing is to deploy each game modifier on each game client so that any dormant data can be revealed. As a result, in FPS, the whole team of players can oversee any movement of any enemy player anywhere.

### 5.4. Manipulating Animation Data

Animation poses and animation state machines can also be manipulated. Animation data are synchronized among clients on the network; thus, ghost movements can be seen by other players if a runner’s animation on the local client has been locked. 

Additive blending is a form of more natural animation blending that is used by most 3D games. The transformation “localPose” is an affine transformation relative to the parent joint, which can be replaced by: quaternion rotation; Vector3 translation; float scale. The animation poses at any moment are interpolated among keyframes, which separately and linearly interpolate the rotation, translation, and scale, instead of “localPose”. Algorithms A3 and A4 in Appendix A can be used to manipulate the animation data.

### 5.5. Manipulating Physics Data

The physics system is an important component in games, which is responsible for simulating reality. However, some games (FPS) can even actively change the physics metrics for more enjoyment in a specific scenario, such as earth gravity decrement, so that the characters will drop down more slowly. Therefore, gravity data can be reverse-manipulated, and the related functions (“applyForce”, “applyTorque”, and “applyImpulse”) can be hooked.

Another supernatural power that can be enabled by reverse data modification is to let players see objects through smoke or fog. Three-dimensional games use functions such as setRenderState (Enable_AlphaBlend) and setRenderState (Enable_fog) to blend objects in and change the screen’s final colors:

(9) $$\mathrm{colors}\;=\left(1-\frac{1}{e^{fog\_density\times distance\_obj}}\right)\times \mathrm{FogTexture}\;\;+\frac{1}{e^{fog\_density\times distance\_obj}}\times \mathrm{Frame}.$$

Furthermore, these games use occlusion culling to remove occluded objects via a z-buffer in the rendering pipeline. There is still a function, setRenderState (Enable_Z-Test), that waits to be hooked behind this usage. 

An axis-aligned bounding box (AABB, which is perfect for rendering tanks in RTSG), an oriented bounding box, and a bounding sphere are most commonly used for intersection detections among objects. A bounding sphere can be used for preliminary detection, and its parameters can be reverse-modified:

(10) $$\left\{\begin{array}{c} \mathrm{radius}=\frac{\sqrt{{\left(x_{max}-x_{min}\right)}^{2}+{\left(y_{max}-y_{min}\right)}^{2}+{\left(z_{max}-z_{min}\right)}^{2}\;}}{2}\\ x_{center}=\frac{x_{max}-x_{min}}{2}\\ y_{center}=\frac{y_{max}-y_{min}}{2}\\ z_{center}=\frac{z_{max}-z_{min}}{2} \end{array}.\right.$$

### 5.6. Manipulating Event Data 

Generally, a random generator produces a deterministic random sequence; therefore, it is called a pseudo-random generator. If a game needs random events, such as explosion rates, the same random sequence needs to be assured among network clients. Furthermore, new events should not be sent when events are being handled, to avoid an event feedback loop. The chain of responsibility pattern and the observer pattern are used in the event system, as Algorithm A5 in Appendix A shows. 

## 6. Experiment and Evaluations

Simulated environments have no correlation with real game environments in terms of huge virtual addresses, real stack traces, real function import tables, etc. Thus, two games that have been installed on a computer with a 2.5 GHZ intel i7 CPU and 16 G of memory are used in this paper for experiments and evaluations. 

Real software is used to present the effectiveness of our method, instead of simulation experiments. The two games are suitable for multiple players and have already been distributed on a public platform. Our method is effective in the following scenarios:

1. Physics data are manipulated so that a scout car is invincible in RTSG, as shown in Section 5.5.
2. Sight data are manipulated so that more entities can be seen in RTSG, as shown in Section 5.2 and Section 5.3.
3. Animation data are manipulated so that the player always “keeps his head down”, as shown in Section 5.4.
4. Aim data are manipulated so that auto-aim is enabled in FPS, as shown in Section 5.2.

## 7. Conclusions

We can imagine the malicious data manipulations that might occur in telemedicine, automatic driving, or other IoT applications, and how terrible the consequences would be. To demonstrate the new, hidden dangers in the new era of 5G, the detailed reverse process is elaborated using real scenarios. To address the three challenges above, (1) the base virtual addresses of software objects can be changed frequently for safety, (2) prior knowledge of reverse is not considered, and (3) the system memory values of some target objects are changed with extreme speed, and a novel reverse methodology (MK) is proposed. In this paper, we recovered the control flow and data flow by analyzing the game functions and virtual memory, then MK reverse-manipulated the class members based on our knowledge of 3D video game development, which is efficient when dealing with the above challenges. The novelties of our work include: (1) explaining the meaning of “seeing is believing” for distributed software with network synchronization; (2) how to achieve “seeing is not believing”; (3) identifying where and when to reverse-manipulate the data. Furthermore, the general architecture of multi-player online 3D video games is presented using high-level program codes (forward perspective) and disassembly codes (reverse perspective). The program-slicing process of game logic, the rendering pipeline, and data synchronization are also presented. In our future work, we will propose counter-methods to decrease the dangers mentioned in this paper.

## Figures and Tables

**Table 1:** A summary of reviewed papers suitable for the reverse modification of binary code and data.

Paper	Algorithm Type	Advantage	Disadvantage
[3,4]	dependency relations discover	static and dynamic program slicing for the target codes	program codes must be obtained previously
[5,6,7,8,9,10,11]	symbolic execution	find all feasible program paths for the target data	not scalable for large programs with unbounded loop iterations
[12,13,14,15,16]	taint analysis	analyze program information flow for the target data	restricted by the problem of hidden taint propagation
[17,18,19,20,21,22,23,24,25,26,27,28]	deep learning	deep learning inference can be used for finding the target data	lack of explainability and long duration of training
[29,30,31,32]	deep reinforcement learning	change classification problems to policy optimization problems	need a large number of simulations
[33,34,35]	integrate logical knowledge into deep learning	improve explainability	longer duration of training

## Data Availability

Not applicable.

## Appendix A

**Algorithm A1:** Target objects filter (assembly codes).
push eax push ebx push ecx cvtss2si eax, [1BCB28F0] // Obtain coordinate X of camera cvtss2si ebx, [1BCB28EC] // Obtain coordinate Z of camera cvtss2si ecx, [esi] // Obtain coordinate X of a target object sub eax, ecx cmp eax, 28 jg @outrange cmp eax, −28 jl @outrange cvtss2si ecx, [esi+4] // Obtain coordinate Z of the target object sub ebx, ecx cmp ebx, 20 jg @outrange cmp ebx, −20 jl @outrange mov [x],esi @outrange: pop eax pop ebx pop ecx

**Algorithm A2:** Target objects filter.
Class Plane Vector3 normal // point-normal form: Dot(normal, u) + d = 0 float d Plane* next bool FrontOrBack(Vector3 u) return Dot(normal, u) + d > 0 ? 1:0 bool InRange (GameObject &go, Plane& plain) if plain == null return true else switch plain.FrontOrBack (go.positon) case 1 return false case 0 return InRange (go.positon, plain.next)

**Algorithm A3:** Animation skinning.
Class Joint Matrix44 Bind // bind pose byte ParentIndex Class Skeleton Joint joints[n] Mesh mesh[i] Class Mesh SkinnedVertices vertices[j] Class SkinnedVertices float Position [3] float Normal [3] float Texture_u, Texture_v byte JointIndex [2] // at most 2 joints affect a vertex float JointWeight [2] Vector3 Skinning (JointPose Pose0, JointPose Pose1) v0 = JointWeight [0]× JointIndex [0].bind × Pose0.localPose v1 = JointWeight [1]× JointIndex [1].bind × Pose1.localPose return v_Model^Bind × (v0 + v1) Class JointPose Matrix44 localPose Class AnimationClip Skeleton* skeleton float FPS bool isLoop JointPose jointPose[m] // m key frames

**Algorithm A4:** The animation state machine.
Class StateMachine State * activeState List<State> states void Update(GameObject& go) if activeState.transitions.atransition.IsValid() activeState.OnExit(go) activeState = validTransition. GetNextState(go) activeState.OnEnter(go) else activeState.OnUpdate(go) Class State List<Transition> transitions virtual void OnEnter (GameObject& go) virtual void OnExit (GameObject& go) virtual void OnUpdate (GameObject& go) Class Transition virtual State * GetNextState(GameObject& go) virtual bool IsValid() // return true when the condition is satisfied Class GroundState: State // hierarchical state machine Class RunState: GroundState virtual void OnEnter (GameObject& go) Go.SetAnimationClip(RUN)

**Algorithm A5:** OnEvent, including all events handlers.
Partial Class CombatUnit: GameObject bool OnEvent(Event& event) if Base::OnEvent(event) return true switch event.type case explosion return eventQueue.Dispatch(event, riders) // Chain of responsibility case fire Fire() // handle the event by itself return true default return false // cannot handle the event
